# Adjuvant-Mediated Differences in Antibody Responses to Computationally Optimized Hemagglutinin and Neuraminidase Vaccines

**DOI:** 10.3390/v15020347

**Published:** 2023-01-25

**Authors:** Kaito Nagashima, Nada Abbadi, Ved Vyas, Abigail Roegner, Ted M. Ross, Jarrod J. Mousa

**Affiliations:** 1 Center for Vaccines and Immunology, College of Veterinary Medicine, University of Georgia, Athens, GA 30602, USA; 2 Department of Infectious Diseases, College of Veterinary Medicine, University of Georgia, Athens, GA 30602, USA; 3 Florida Research and Innovation Center, Cleveland Clinic, Port Saint Lucie, FL 34987, USA; 4 Department of Infection Biology, Lerner Research Institute, Cleveland Clinic, Cleveland, OH 44195, USA; 5 Department of Biochemistry and Molecular Biology, Franklin College of Arts and Sciences, University of Georgia, Athens, GA 30602, USA

**Keywords:** influenza, vaccine, COBRA, hemagglutinin, neuraminidase, adjuvant, antibody

## Abstract

Computationally optimized broadly reactive antigens (COBRAs) are a next-generation universal influenza vaccine candidate. However, how these COBRAs induce antibody responses when combined with different adjuvants has not previously been well-characterized. Therefore, we performed in vivo studies with an HA-based H1 COBRA, Y2, and an NA-based N1 COBRA, N1-I, to assess this effect for the H1N1 subtype. We tested the adjuvants AddaVax, AddaS03, CpG, and Alhydrogel. AddaS03 performed the best, eliciting high IgG titers and hemagglutination inhibition (HAI) activity for Y2 immunizations. Interestingly, serum antibody epitopes were relatively similar across adjuvant groups. Moreover, following N1-I immunization with these adjuvants, AddaS03 also elicited the highest IgG and neuraminidase inhibition (NAI) titers against the 2009 pandemic virus, A/California/07/2009 (A/CA/09). These results inform adjuvant selection efforts for H1 and N1 COBRA HA and NA antigens in a mouse model.

## 1. Introduction

Influenza poses a significant health burden worldwide [1]. It is responsible for annual epidemics, for which the only major countermeasure is the seasonal influenza vaccine. However, due to the highly mutable nature of influenza virus, seasonal influenza vaccines have variable and short-lived effectiveness, requiring annual vaccination. In an effort to provide broadened and longer-lasting protection, a next-generation vaccine design platform, termed COBRA, or computationally optimized broadly reactive antigen, was developed [2,3,4]. COBRAs can broaden the antibody response against the surface glycoproteins hemagglutinin (HA) and neuraminidase (NA). This platform incorporates wild-type HA or NA sequences from selected time periods and antigenic spaces to produce a COBRA immunogen that elicits enhanced antibody breadth. These COBRA vaccines were designed for the influenza viruses that cause disease in humans, influenza A viruses (IAVs) and influenza B viruses (IBVs). In both naïve and pre-immune animal models, immunization with COBRA HAs and NAs typically produces protective and broadly acting antibody responses against a wide range of viruses [4,5,6].

H1N1 subtype viruses circulate every year and comprise one component of the seasonal influenza vaccine, alongside H3N2 and IBVs [7]. Viruses of this subtype were in circulation from 1918 to 1957, disappearing from 1957 to 1977, and then re-emerging and remaining for every season since [8]. The 2009 swine influenza pandemic, resulting from a reassortment event between avian, swine, and human influenza viruses, led to an antigenic shift, since which antigenically similar viruses have been in circulation [8]. Several HA antibody epitopes have also been characterized in the immunodominant head domain and the immunosubdominant stem domain. The receptor-binding site (RBS) is a conserved target of certain head-binding antibodies that prevent sialic acid binding and host cell attachment [9]. In addition, the lateral patch epitope is another conserved region on the head domain [10,11]. Within the more conserved stem domain, the central stem epitope is a target of group 1-specific, broadly reactive antibodies including CR6261 [12]. Antibodies that bind the membrane-proximal region of the HA at the anchor epitope have been identified as well [13,14]. Whereas the HA component of each virus varies substantially across strains, the NA component remains relatively stable, accruing mutations only occasionally [15]. Current seasonal vaccines are standardized by HA content but not by NA content, despite the more conserved nature of this glycoprotein. Furthermore, anti-NA titers have been observed to be a useful correlate of protection [16,17]. Likewise, employing an NA-based universal vaccine to supplement an HA-based vaccine may afford longer-term protection.

Adjuvants are critical components of several licensed vaccines and drive enhanced immune responses, particularly for subunit vaccines [18]. Adjuvants can act through several pathways to exert their effects, which include polarization of the overall immune response and skewing of antibody epitopes [18]. Moreover, they can alter B cell receptor affinity and diversity, as has been shown for MF59 [19]. In this study, we aimed to characterize the effect of adjuvant on the antibody response to vaccinations with COBRA immunogens. This involved analysis of HA antibody epitopes, Th1 versus Th2 responses, and antibody functionality for the H1 subtype HA. We also assessed total and functional antibody titers elicited by the N1 subtype of NA of H1N1 viruses for the following adjuvants: AddaVax, AddaS03, CpG ODN 2395, and Alhydrogel. AddaVax and AddaS03, analogs of MF59 and AS03, respectively, are oil-in-water emulsions that enhance somatic hypermutation and affinity maturation [20]. In contrast, CpG is a TLR9 agonist [21], and Alhydrogel is an analog for alum [22], which may operate through the depot effect and adsorption of antigen, eliciting a Th2-skewed response. We found that adjuvants differentially alter total serum titers against the leading H1 COBRA HA candidate Y2 [23], designed from human HA sequences from 2014 to 2016, and N1 COBRA NA candidate N1-I [4], designed from human, avian, and swine sequences from 1990 to 2015. Moreover, the epitope profiles on the A/California/04/2009 pandemic HA were similar across these adjuvants when used in immunizations with Y2. AddaS03 elicited high and broadly functional antibodies when combined with COBRAs in immunizations, suggesting that it may be an ideal adjuvant to supplement with the Y2 and N1-I antigens. Overall, these data suggest that the choice of adjuvant plays a significant role on antibody titers and functional potency following immunization with next-generation influenza vaccines.

## 2. Materials and Methods

Protein production. Trimeric wild-type CA09 HA, Y2 COBRA HA, and chimeric HA cH6/1 composed of the globular head region from H6N1 isolate A/mallard/Sweden/81/2002 and the HA stem region of the 2009 pandemic H1N1 isolate [24], as well as tetrameric N1-I NA ectodomains, were expressed and purified in Expi293F cells following the manufacturer’s protocol as previously described [25]. Collected supernatants containing the HA or NA antigens were purified on a HisTrap Excel column (GE Healthcare, Chicago, IL, USA) following the manufacturer’s recommended protocol. Eluted fractions were pooled, and purified proteins were verified for integrity by probing with an anti-HIS tag antibody (clone J099B12, BioLegend, San Diego, CA, USA) as well as with subtype-specific mAbs via SDS-PAGE and Western blot.

### 2.1. Viruses

The viruses used, A/California/07/2009 (A/CA/09), A/Brisbane/02/2018 (A/BR/18), and A/Guangdong-Maonan/SWL1536/2019 (A/GM/19), were grown for two passages in MDCK London (FR-58, IRR) cells. Viruses were titered by hemagglutination (HA) assays using 1% turkey blood (Lampire, Pipersville, PA, USA).

### 2.2. Animals and Vaccinations

All procedures were reviewed and approved by the University of Georgia Institutional Animal Care and Use Committee (IACUC). For the H1 HA study, 10 six-to-eight-week-old male and female BALB/c mice (Charles River Laboratories, Wilmington, MA, USA) per group were immunized subcutaneously with 20 μg of Y2 COBRA HA, CA09 HA, or phosphate-buffered saline (PBS) adjuvanted with AddaVax, AddaS03, CpG ODN 2395, and Alhydrogel (Invivogen, San Diego, CA, USA), or with PBS as a no adjuvant control. Mice were bled at 27 days post-prime for d27 serum, then immunized at 28 days post-prime with 20 μg of the antigen. At 56 days post-prime, animals were bled for d56 serum, and then euthanized with Avertin.

For the N1-I NA study, five six-to-eight-week-old female BALB/c mice (Charles River Laboratories, Wilmington, MA, USA) per group were immunized intramuscularly with 6 μg of N1-I COBRA HA or PBS adjuvanted with AddaVax, AddaS03, CpG ODN 2395, and Alhydrogel (Invivogen, San Diego, CA, USA), or with PBS as a no adjuvant control. Mice were bled at 27 days post-prime for d27 serum, then immunized at 28 days post-prime with 6 μg of the antigen. At 56 days post-prime, animals were bled for d56 serum, and then euthanized with Avertin.

### 2.3. Enzyme-Linked Immunosorbent Assays (ELISAs)

For total and chimeric H6/1 (cH6/1) IgG ELISAs, 384-well plates (Greiner Bio-One, Monroe, NC, USA) were coated with an antigen diluted to 2 μg/mL in PBS at 4 °C overnight. The plates were washed once with water and then blocked with 2% blocking buffer (PBS  +  2% nonfat dry milk (Bio-Rad, Hercules, CA, USA)  +  2% goat serum  +  0.05% Tween-20) for 1 h at room temperature. The plates were washed three times with water, and 25 μL of diluted mouse serum was added. Sera were serially diluted three-fold in 1% blocking buffer from a 1:50 initial dilution for 12 total dilutions. The plates were incubated at 37 °C for 1 h and then washed three times with water. Goat anti-mouse IgG Fc-AP secondary antibody (Southern Biotech, Birmingham, AL, USA), diluted to 1:4000 in 1% blocking buffer (1:1 dilution of PBS and 2% blocking buffer), was added, and the plates were incubated at room temperature for 1 h. The plates were then washed five times with PBS-T (PBS  +  0.05% Tween 20). p-Nitrophenyl phosphate (PNPP) substrate (ThermoFisher Scientific, Waltham, MA, USA), diluted in substrate buffer (1.0 M Tris  +  0.5 mM MgCl2, pH 9.8) to 1 mg/mL, was added, and the plates were incubated for 1 h and read at 405 nm on a BioTek plate reader (Agilent, Santa Clara, CA, USA). The area under the curve (AUC) value for each mouse group was determined using GraphPad Prism 9 software (GraphPad, San Diego, CA, USA) using a baseline of 0.3 absorbance units at 405 nm and log_10_-transformed serum dilutions.

For Th1/Th2 ELISAs, to determine the relative abundance of IgG subclasses to Y2 or CA09 HAs, a similar protocol was used for coating and blocking steps. After these steps, plates were washed three times with water, and serum was pooled across male or female mice for each group. Pooled serum was then serially diluted three-fold in 1% blocking buffer from a 1:50 dilution for 12 dilutions and added to the ELISA plate and incubated at 37 °C for 1 h. Plates were washed three times with water, and goat anti-mouse IgG1, IgG2a, IgG2b, IgG2c, or IgG3 Fc-AP secondary antibody (Southern Biotech, Birmingham, AL, USA) diluted to 1:4000 in 1% blocking buffer was added in separate wells and incubated for 1 h at room temperature. The plates were then washed five times with PBS-T. PNPP substrate (ThermoFisher Scientific, Waltham, MA, USA), diluted in substrate buffer to 1 mg/mL, was added, and the plates were incubated for 1 h and read at 405 nm on a BioTek plate reader (Agilent, Santa Clara, CA, USA). The AUC value for each mouse group was determined using GraphPad Prism 9 software (GraphPad, San Diego, CA, USA) using a baseline of 0.3 absorbance units at 405 nm and log_10_-transformed serum dilutions.

### 2.4. Competition ELISAs

Human monoclonal antibodies (mAbs) used in competition ELISAs were isolated and characterized previously [14]. 384-well plates (Greiner Bio-One, Monroe, NC, USA) were coated with CA09 HA diluted to 2 µg/mL in PBS at 4 °C overnight. Plates were washed once with water and blocked with 2% blocking buffer for 1 h at room temperature. Plates were washed three times with water. Pooled mouse serum was three-fold serially diluted from a 1:10 dilution in 1% blocking buffer, and then human mAbs specific for head and stem domain epitopes diluted in 1% blocking buffer were added in a 1:1 ratio. Alternatively, 1% blocking buffer was added to mAbs in a 1:1 ratio for the mAb only control. Next, 25 µL of the serum/mAb or the 1% block/mAb mixture was added to the plate and incubated at 37 °C for 1 h. Plates were then washed three times with water, and 25 µL of goat anti-human IgG Fc, multispecies SP ads-AP (Southern Biotech, Birmingham, AL, USA) diluted to 1:4000 in 1% blocking buffer, was added and then incubated for 1 h. Plates were washed five times with PBS-T, and then PNPP substrate diluted to 1 mg/mL in substrate buffer was added, incubated for 1 h, and read at 405 nm on a BioTek plate reader (Agilent, Santa Clara, CA, USA). Competition was calculated as the ratio of signal from a given serum dilution with mAb to the signal of the corresponding dilution in the mAb only control. Low competition was defined as a ratio between 0 and <0.330, intermediate competition between ≥0.330 and <0.660, and high competition between ≥0.660 and 1.

### 2.5. Hemagglutination Assays (HAs)

First, 50 µL of virus was diluted two-fold in PBS from a 1:2 dilution for 50 µL total for 11 dilutions. Turkey whole blood in Alsevers’ solution (Lampire, Pipersville, PA, USA) was washed three times with PBS and diluted to a 1.0% concentration in PBS. Next, 50 µL of the 1.0% turkey red blood cells was added per well. Plates were read 45 min after the addition of 1.0% turkey red blood cells. The well with the highest virus dilution that did not cause turkey red blood cells to drip (i.e., cause hemagglutination) was determined to be the HA titer.

### 2.6. Hemagglutination Inhibition Assays (HAIs)

Serum was treated with receptor-destroying enzyme II (RDE II, Denka Seiken, Tokyo, Japan) to remove background hemagglutination activity. Briefly, one volume of serum was added to three volumes of RDE II in PBS and incubated at 37 °C overnight. The following day, the treated serum was heat-inactivated at 56 °C for 45 min, allowed to cool to room temperature, and then six volumes of PBS were added. Influenza viruses were titered to eight HAUs (hemagglutination units) per mL. Next, 50 μL of RDE-treated serum was added to the first well of a 96-well V-bottom plate (ThermoFisher Scientific, Waltham, MA, USA) and diluted two-fold in PBS for 25 μL total for 11 dilutions. The virus, titered to eight HAUs per mL, was added in a 1:1 ratio to each serum dilution, and each well was mixed and incubated for 20 min at room temperature. Following this, 50 μL of 1.0% turkey red blood cells was added per well following three washes of turkey whole blood in Alsevers’ solution (Lampire, Pipersville, PA, USA) with PBS as described in the HA methods. Plates were read 45 min after the addition of 1.0% turkey red blood cells. The well with the highest serum dilution containing dripped turkey red blood cells (i.e., showing lack of hemagglutination) was determined to be the HAI titer. Although the WHO and the European Committee for Medicinal Products have defined a 1:40 titer to be seroprotective in humans [26], a more stringent 1:80 titer was defined to be seroprotective, similar to the cutoff determined previously [27].

### 2.7. Enzyme-Linked Lectin Assays (ELLAs)

To determine A/California/07/2009 NA activity, high-binding flat-bottom 96-well plates (Greiner Bio-One, Monroe, NC, USA) were coated with 100 μL of 25  μg/mL fetuin (Sigma-Aldrich, St. Louis, MO, USA) at 4 °C overnight. Influenza virus A/California/07/2009 was diluted in sample diluent (PBS, 1% BSA, 0.5% Tween-20) to an initial dilution of 1:10 and then serially diluted two-fold for 11 dilutions. A negative-control column was included containing 100 μL of only the sample diluent. Fetuin plates were washed three times with PBS-T (PBS  +  0.05% Tween 20). Next, 50 μL of serially diluted virus was added to the fetuin-coated plate containing 50 μL of the sample diluent in duplicate. Plates were incubated for 18 h at 37 °C and 5% CO_2_. After incubation, plates were washed six times in PBS-T, and 100 μL of peanut agglutinin-HRPO (Sigma-Aldrich, St. Louis, MO, USA) diluted 1000-fold in conjugate diluent (PBS, 1% BSA) was added. Plates were incubated at RT for 2 h. Plates were washed three times in PBS-T, and 100 μL (500 μg/mL) of o-phenylenediamine dihydrochloride (OPD) (Sigma-Aldrich, St. Louis, MO, USA) in phosphate-citrate buffer (Sigma-Aldrich, St. Louis, MO, USA) was added to the plates. Plates were incubated in the dark for 10 min at RT. The reaction was stopped with 100 μL of 1 N sulfuric acid. The absorbance was read at 490 nm on a BioTek plate reader (Agilent, Santa Clara, CA, USA). The dilution of the virus needed to achieve 90 to 95% NA activity was determined and used for subsequent NA inhibition ELLAs.

### 2.8. Neuraminidase Inhibition Assays (NAIs)

Mouse sera were heat inactivated at 56 °C for 1 h. The serum was serially diluted five-fold in the sample diluent from the 1:100 initial dilution for 10 dilutions. A negative-control column (no serum or virus) was included containing 100 μL of only the sample diluent. A virus only column was also included. Then, 50 μL of duplicate dilutions was added to fetuin plates and 50 μL of the virus diluted to 90 to 95% NA activity in the sample diluent was added to the plate. Plates were incubated for 18 h at 37 °C and 5% CO_2_, after which they were processed as described above with the ELLAs. Endpoint titers were identified as the highest serum dilution that resulted in at least 50% inhibition of the maximum signal.

### 2.9. Quantification and Statistical Analysis

One-way ANOVA was used to compare between vaccination groups using GraphPad Prism 9 software (GraphPad, San Diego, CA, USA). Statistical significance was defined as follows: *, *p* ≤ 0.05; **, *p* ≤ 0.01; ***, *p* ≤ 0.001; ****, *p* ≤ 0.0001.

## 3. Results

### 3.1. Adjuvanted Vaccination with Y2 Alters Serum Antibody Titers to the Y2 COBRA HA

**Figure 1:**
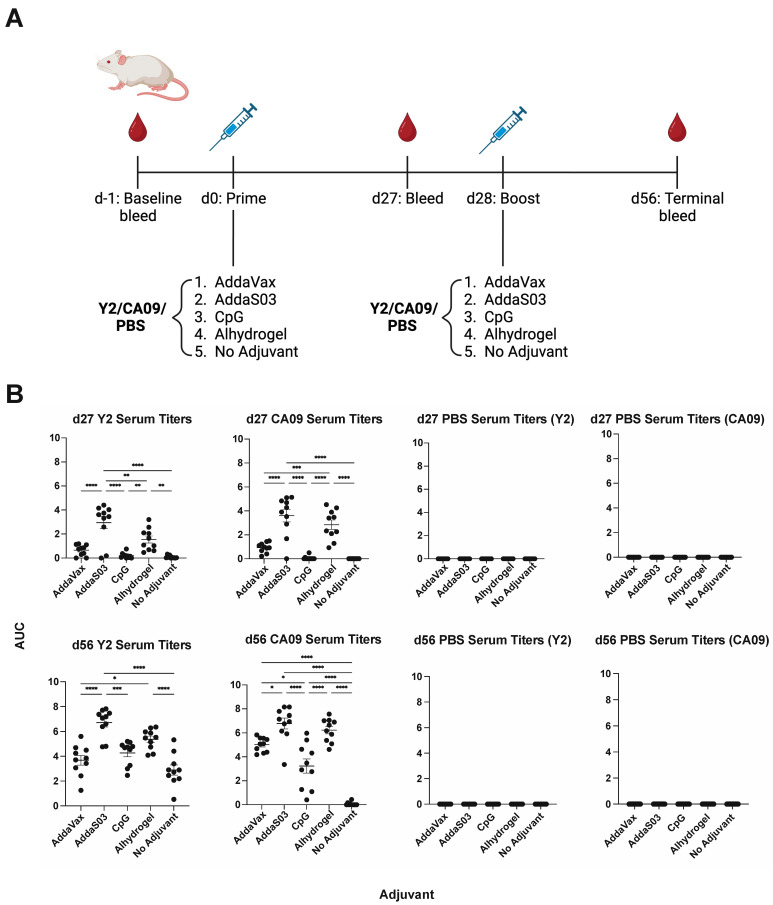
H1 HA immunization schedule and serum IgG titers after adjuvanted vaccination with Y2 and CA09 HAs. (**A**) Overall immunization scheme of the H1 HA study. Time points for bleeds and vaccinations are shown. (**B**) Titers of IgG antibodies in sera from vaccinated mice, determined by ELISA, are shown for 27 days post-prime after one immunization (d27, top) for all groups against the homologous immunizing HA. Titers for both HAs are shown for the PBS immunization control. Those at 56 days post-prime (28 days post-boost) are shown on the bottom. Titers are represented as area under the curve (AUC) values, where a minimum baseline absorbance at 405 nm of 0.3 was considered a positive binding signal. *, *p* ≤ 0.05; **, *p* ≤ 0.01; ***, *p* ≤ 0.001; ****, *p* ≤ 0.0001. One-way ANOVA was used for statistical comparisons between groups.

To determine the serum IgG response to adjuvanted wild-type 2009 pandemic A/California/04/2009 (CA09) HA or COBRA HA vaccination, we used a prime-boost regimen in mice (Figure 1A). Animals were immunized with either the Y2 COBRA HA or the CA09 HA. Four adjuvants were used—AddaVax, AddaS03, CpG ODN 2395 and Alhydrogel—or no adjuvant was used as a control. We then evaluated titers against the homologous immunizing antigen by ELISA. At 27 days post-vaccination, we found that AddaS03 elicited the highest antibody titers to their respective immunizing antigens, followed by Alhydrogel, AddaVax, and CpG (Figure 1B). This pattern was consistent across both Y2 and CA09 immunizations. This trend was conserved at 56 days post-vaccination after a single boost, where AddaS03 and Alhydrogel elicited the highest titers. Notably, we found that the no adjuvant control elicited minimal titers for the CA09 HA after two immunizations, whereas all adjuvanted groups showed detectable serum IgG titers. In comparison, the no adjuvant control for the Y2-immunized animals elicited significant titers to their respective antigens after a prime and a boost, suggesting Y2 is more immunogenic in the BALB/c mouse background. As expected, adjuvanted groups generally showed improved titers relative to the no adjuvant control, and no titers were observed for PBS-immunized animals. Therefore, we found that formulation of the HA antigen with various adjuvants elicited elevated levels of serum IgG relative to the no adjuvant control, and AddaS03 was superior in this respect.

**Figure 2:**
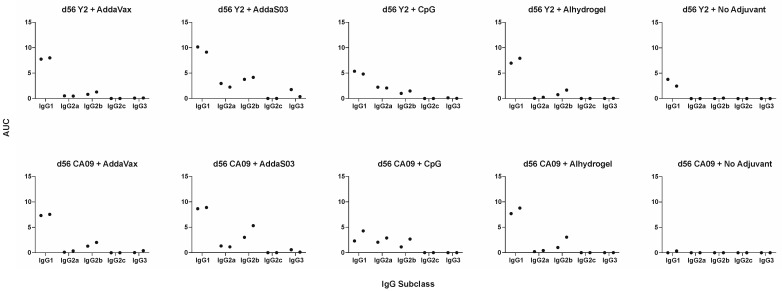
Th1/Th2 ELISAs at d56 after adjuvanted vaccination with Y2 and CA09 HAs. IgG subclass-specific serum titers, determined by ELISA, are shown for male and female pooled sera at d56 as AUC values. Coating antigens were the same HA as used for vaccination, The left point for each IgG subclass corresponds to male mice serum titers and the right point to female mouse serum titers. A minimum baseline absorbance at 405 nm of 0.3 was considered a positive binding signal.

Adjuvants can alter the overall immune profile following immunization, biasing it towards a Th1, Th2, or balanced Th1/Th2 response [28]. To evaluate this effect in mice, we measured the relative concentrations of IgG subclasses within the serum (Figure 2). We pooled male and female mouse serum from each group and evaluated terminal titers against the homologous HA for subclasses IgG1, IgG2a, IgG2b, IgG2c, and IgG3. We found that the choice of adjuvant alters the Th1/Th2 ratio, suggesting changes within the overall immune profile, with most adjuvants eliciting primarily a Th2-skewed response. Overall, trends were similar between Y2 and CA09 HA immunizations across adjuvants. AddaVax elicited a Th2-biased response, where most serum IgG antibodies were of the IgG1 subclass, and a minority were of the IgG2b subclass. AddaS03 elicited both IgG1 and IgG2b antibodies, but IgG1 titers were nonetheless elevated compared to IgG2b in comparison to the Y2 no adjuvant control. CpG was associated also with a balanced response, with both IgG1 and IgG2 antibodies observed in similar ratios, albeit at lower overall titers. Expectedly, Alhydrogel, a known Th2-skewing adjuvant, elicited a Th2-biased response with most antibodies being of the IgG1 subclass. For the Y2 no adjuvant control, we detected IgG1 antibodies only, which is consistent with the inherent Th2-skewed response seen with the BALB/c background.

### 3.2. Serum Antibody Epitopes Are Similar between Adjuvants following Y2 Immunization

**Figure 3:**
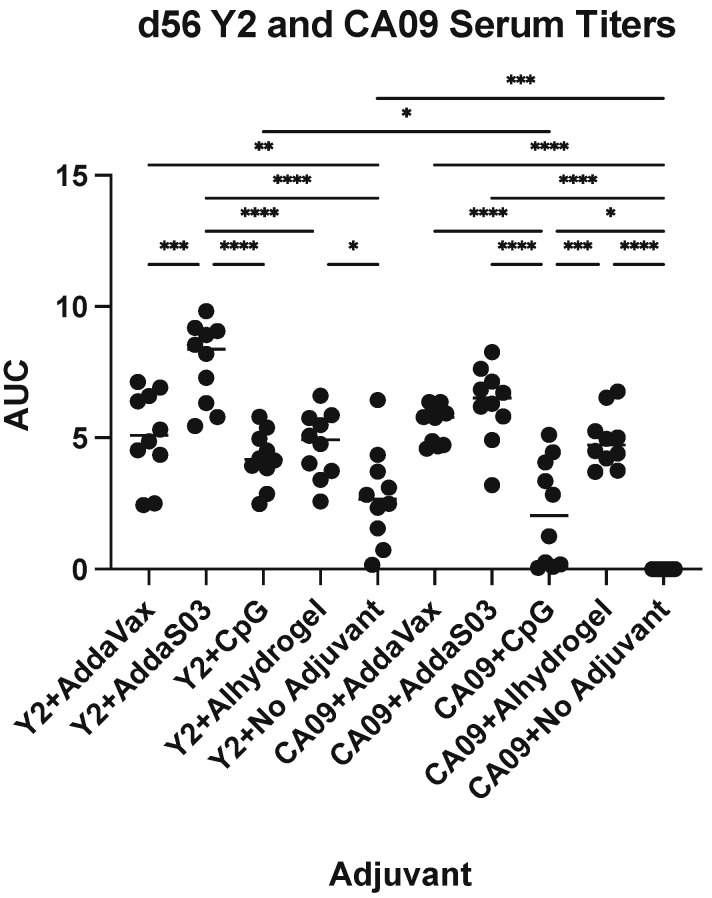
cH6/1 HA IgG titers at d56 after adjuvanted vaccination with Y2 and CA09 HAs. Serum IgG titers, determined by ELISA, are shown as AUC values for both Y2- and CA09 HA-immunized mouse groups. A minimum baseline absorbance at 405 nm of 0.3 was considered a positive binding signal. *, *p* ≤ 0.05; **, *p* ≤ 0.01; ***, *p* ≤ 0.001; ****, *p* ≤ 0.0001. One-way ANOVA was used for statistical comparisons between groups.

In addition to total serum IgG responses to the immunizing HA, we also determined the serum IgG response to the H1 HA stem domain (Figure 3). The stem domain is more conserved than the head domain across influenza strains and serves as a target for some universal vaccine candidates, such as the chimeric HA (cHA) approach [29]. To evaluate antibodies against this domain, we evaluated binding to the cH6/1 cHA construct [24,30]. The cH6/1 cHA contains an exotic H6 subtype head genetically fused to the H1 subtype CA09 HA stem. Four weeks after boost, we found that there were significant titers for all adjuvanted groups for both Y2 and CA09 HA immunizations. The differences between adjuvants closely mirrored those for serum IgG titers against the Y2 and CA09 HA antigens. AddaS03 elicited the highest cH6/1-binding IgG responses, followed by Alhydrogel and AddaVax, with CpG eliciting the lowest titers of all adjuvanted groups. For Y2 immunizations, we detected antibodies against the cH6/1 HA as well, potentially suggesting that stem antibodies may be elicited. We also compared titers between Y2- and CA09 HA-immunized animals against the cH6/1, finding statistically increased titers for Y2 relative to CA09 HA for the CpG-adjuvanted and no adjuvant groups. This implied that to some extent, Y2 is more immunogenic than the CA09 HA. Of note, it is possible that antibodies targeting non-HA-specific regions, such as the trimerization domain or the His-tag, could be contributing to this antibody response. Nonetheless, the observation of cH6/1 HA-specific serum antibodies may suggest that the adjuvants evaluated here may amplify stem-specific responses to some extent.

**Figure 4:**
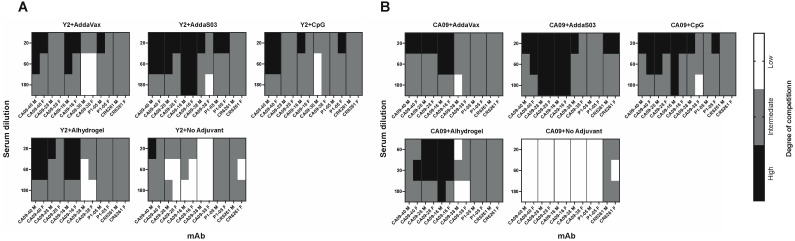
Epitope mapping of d56 sera against the CA09 HA from Y2- and CA09 HA-immunized mice. Competition ELISAs were used to determine the HA epitopes bound by mouse serum for Y2- and CA09 HA-immunized mice. Sera from pooled mice were competed with human mAbs specifically against distinct HA head and stem epitopes. The degree of competition for serially diluted mouse sera with the indicated human mAbs is shown as a heat map for final serum dilutions of 1:20, 1:60, and 1:180 for these groups for (**A**) Y2-immunized mice and (**B**) CA09 HA-immunized mice. M, male pooled serum; F, female pooled serum.

To assess the specific epitopes targeted by serum from adjuvanted Y2 immunizations, we performed competition ELISAs against the wild-type CA09 HA (Figure 4 and Figure S1). We assessed the competition of pooled terminal serum against a panel of previously characterized human monoclonal antibodies (mAbs) that bind both the head and stem domains, including the recently characterized anchor epitope [14]. Serum competition was observed as a decrease in human mAb-binding signal relative to the mAb only control (Figure S1). We did not see any competition for the CA09 HA-immunized no adjuvant control, as expected due to minimal serum titers after a prime and boost. Otherwise, however, we found similar trends between Y2- (Figure 4A) and CA09 HA-immunized groups (Figure 4B). For most adjuvanted groups, a large proportion of head domain-binding antibodies competed with human mAb CA09-16, which binds near the RBS. This was most pronounced in AddaS03- and Alhydrogel-adjuvanted mice. For the Y2 no adjuvant control, we also saw moderate competition of serum with this antibody. In addition, we saw some competition from the sera of all groups with CA09-40, which also binds near the RBS. Lateral patch-binding serum antibodies were also detected in Y2- and CA09 HA-immunized mouse sera as seen by competition with mAb CA09-28, again being most pronounced for AddaS03- and Alhydrogel-adjuvanted groups. Only low amounts of these antibodies appeared in the no adjuvant Y2 only control. In addition to head-specific serum antibodies, those targeting the stem were also seen for both the central stem and anchor epitopes. Overall, for CA09 HA-immunized groups, all adjuvanted groups showed low to intermediate competition with the central stem-binding antibody CR6261, and either similar or slightly higher levels of competition with the P1-05 anchor epitope-binding antibody. Similar results were seen with Y2 HA-immunized mice, where stem-binding antibodies appeared to be immunosubdominant to head-binding antibodies. Again, the extent of anchor-binding antibodies was either similar to, or slightly higher than, those of central stem-binding antibodies, as seen for the AddaS03- and AddaVax-adjuvanted groups. The Y2 no adjuvant group showed similar, if not slightly lower, competition with these stem epitopes than adjuvanted groups. In general, the epitopes targeted between the head and stem domains were similar across all adjuvanted groups, despite differences in total serum titers. In addition, RBS-binding antibodies, and to a lower extent, anchor epitope-binding antibodies, were generally enhanced by the addition of adjuvant.

### 3.3. Adjuvants Confer Altered HAI Antibody Breadth Following Y2 Immunization

**Figure 5:**
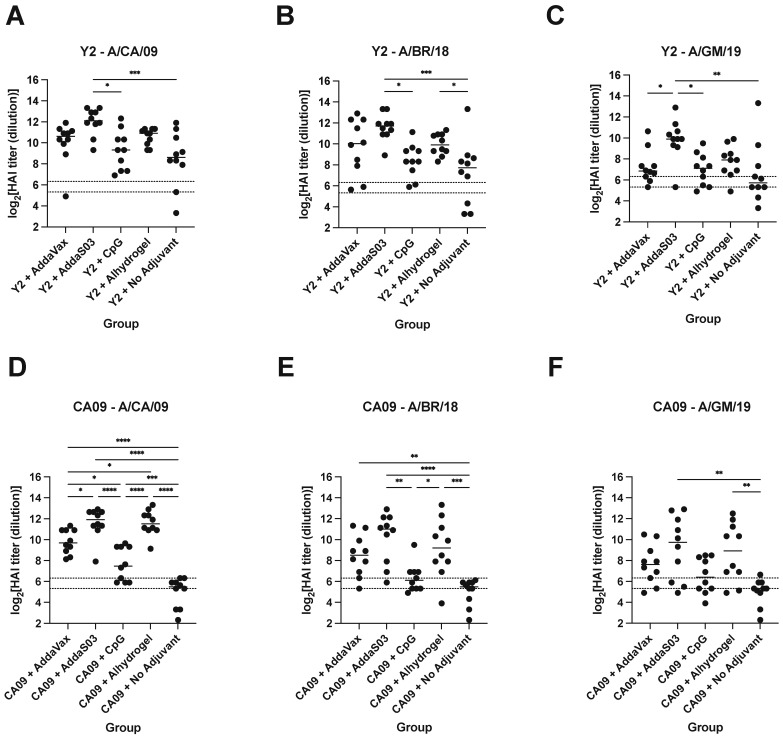
HAI titers of d56 sera from Y2- and CA09 HA-immunized mice. HAI titers against a panel of recent H1N1 viruses are shown for (**A**–**C**) Y2- and (**D**–**F**) CA09 HA-immunized animals. Titers are represented as log_2_-transformed reciprocal dilutions. The lower dotted line represents a titer of 1:40 and the upper line a titer of 1:80, which we defined as the threshold for seroprotection in this study. A/CA/09, A/California/07/2009; A/BR/18, A/Brisbane/02/2018; A/GM/19, A/Guangdong-Maonan/SWL1536/2019. *, *p* ≤ 0.05; **, *p* ≤ 0.01; ***, *p* ≤ 0.001; ****, *p* ≤ 0.0001.

To determine whether the addition of adjuvant could alter the functionality of serum antibodies, we performed HAI assays against a panel of recent 2009 pandemic-like H1N1 viruses (Figure 5). We saw similar trends between HAI titers and ELISA serum IgG titers across adjuvants, where AddaS03 elicited the highest titers, followed by AddaVax and Alhydrogel, with CpG generally inducing the lowest titers of adjuvanted groups. All groups developed seroprotective titers (HAI titer >1:80) against the A/California/07/2009 (A/CA/09) virus for Y2-immunized animals (Figure 5A). In the Y2 no adjuvant control, some individuals developed higher HAI titers against A/CA/09 than in adjuvanted groups, which may reflect the relatively high immunogenicity of Y2. In contrast, lower but seroprotective titers were observed only with adjuvanted groups for the CA09-immunized mice, but not with the non-adjuvanted CA09-immunized animals against A/CA/09 (Figure 5D). With respect to antibody breadth, it appeared that Y2 immunization with AddaS03 elicited the highest HAI breadth against all three viruses, where titers significantly above 1:80 were observed against A/Brisbane/02/2018 (A/BR/18) and A/Guangdong-Maonan/SWL1536/2019 (A/GM/19) (Figure 5B,C). Y2 immunization adjuvanted with Alhydrogel also appeared to elicit statistically significantly increased titers against A/BR/18 relative to the no adjuvant control (Figure 5B). Y2+CpG and Y2+AddaVax, while eliciting HAI titers above the threshold for seroprotection against A/GM/19, overall showed lower titers compared to the Y2+AddaS03 adjuvant group for this virus (Figure 5C). Of CA09-immunized mice, those receiving AddaS03 adjuvant developed the highest HAI titers against the A/BR/18 and A/GM/19 viruses (Figure 5E,F). In addition, Alhydrogel-adjuvanted CA09 immunization appeared to confer broad HAI activity against all of the H1N1 viruses relative to the no adjuvant control (Figure 5D–F).

### 3.4. Adjuvanted Vaccination with the N1-I COBRA Elicits Differential Serum IgG Titers and NAI Titers against a 2009 Pandemic Virus

**Figure 6:**
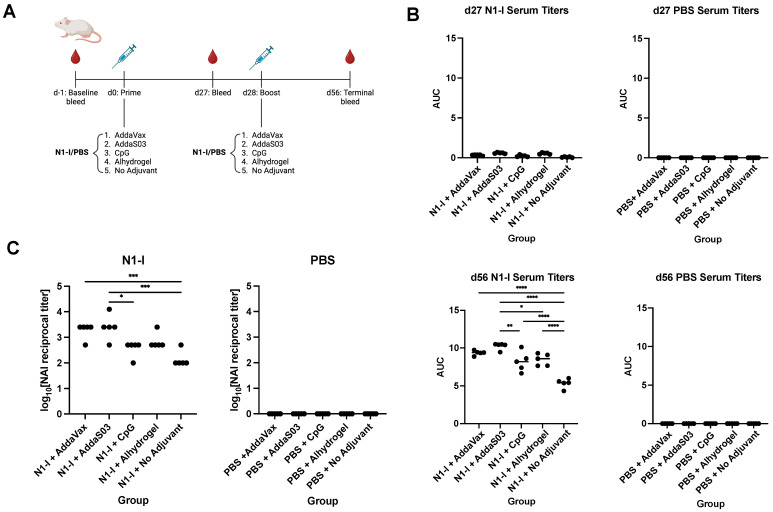
N1-I NA immunization schedule, serum IgG titers, and NAI titers after vaccination. (**A**) Immunization scheme of the N1-I NA study. (**B**) d27 and d56 serum IgG titers, determined by ELISA, against the N1-I NA COBRA. Titers are shown as AUC. (**C**) Terminal NAI titers after vaccination against A/CA/09, shown as log_10_-transformed reciprocal dilutions. NAI titers were determined as the serum dilution that provided 50% inhibition of NA activity. A minimum baseline absorbance at 405 nm of 0.3 was considered a positive binding signal. *, *p* ≤ 0.05; **, *p* ≤ 0.01; ***, *p* ≤ 0.001; ****, *p* ≤ 0.0001. One-way ANOVA was used for statistical comparisons between groups.

Although HA has been the primary focus of universal influenza vaccines, neuraminidase (NA) has been shown to elicit protection as well [31]. Likewise, we assessed the effect of adjuvant on serum IgG titers against the N1-I COBRA, an NA-based universal vaccine candidate [4] (Figure 6A). The same panel of adjuvants were used in a prime-boost regimen with N1-I. At 27 days following prime, minimal titers were observed for N1-I-immunized mice, and none for the PBS control group. Following a boost, however, serum IgG titers mirrored those seen with Y2 immunizations, although differences between adjuvants were less pronounced (Figure 6B). AddaS03 elicited the highest serum IgG titers, whereas AddaVax, Alhydrogel, and CpG elicited titers were somewhat lower. N1-I vaccination without adjuvant elicited notably lower titers than those with adjuvant, as expected. Overall, these data suggest that general trends between these adjuvants may be similar regardless of whether the HA or NA COBRA is used during immunization. Again, supplementing with AddaS03 adjuvant elicited the highest titers of all adjuvanted groups.

NAI assays were performed to assess the functionality of serum antibodies following N1-I vaccination against the A/CA/09 virus (Figure 6C). We found that NAI titers were highest for AddaS03 and AddaVax, followed by Alhydrogel and CpG. Again, these trends are similar to those seen in ELISAs. These results imply that serum antibodies elicited by N1-I are functional and can decrease NA enzymatic activity. Oil-in-water emulsion adjuvants appeared to be more effective than CpG or Alhydrogel in inducing these NA-inhibitory antibodies.

## 4. Discussion

In this study, we assessed the effect of adjuvant on serum antibody responses against both an HA COBRA, Y2, and an NA COBRA, N1-I, against H1N1 viruses. We found a general trend in total serum titers across both of these antigens for the panel of adjuvants tested. Overall, AddaS03 appeared to elicit the highest IgG titers against both Y2 and N1-I, as well as the broadest and highest functional serum antibody titers as assessed by HAI and NAI, respectively.

Y2 has been shown to elicit robust humoral responses in several animal models and is protective against challenges with recent H1N1 isolates [23]. In our study, we found that the addition of adjuvant was able to increase overall serum IgG titers against this COBRA HA, as expected. However, the degree to which this increased varied based on the adjuvant used. Y2, when combined with AddaS03 and Alhydrogel, appeared to elicit the highest titers, which were apparent following a single vaccination. AddaVax and CpG also elicited significant titers, but differences between these groups and the no adjuvant control did not reach statistical significance. We saw similar trends when the wild-type CA09 HA was adjuvanted, again being highest for the AddaS03 and Alhydrogel groups, followed by AddaVax and CpG. We did not see significant titers at d56 for the non-adjuvanted CA09 HA control in contrast to the Y2 non-adjuvanted control, which may suggest that COBRA HAs possess enhanced immunogenicity relative to wild-type HAs.

We also observed the induction of potential stem domain-specific antibodies, as significant serum IgG titers against the cH6/1 HA were seen following Y2 immunization. Trends between adjuvants were relatively similar to those observed with the homologous immunizing HA, where the addition of AddaS03 enhanced serum titers the most of all adjuvanted groups. We also tested individual serum antibody epitopes by competition ELISAs with human mAbs. These experiments suggested that the addition of adjuvant may skew serum epitopes primarily towards head domain epitopes and somewhat to stem domain epitopes. We observed significant competition with the RBS-specific CA09-16 mAb for all adjuvant groups, as well as to the CA09-28 lateral patch epitope, albeit to a lower extent. Additionally, AddaVax- and CpG-adjuvanted groups showed somewhat similar epitope profiles to one another, characterized by moderate competition with another RBS-proximal head-binding mAb, CA09-40, and CA09-16. All adjuvanted groups showed some competition with CA09-38, a previously uncharacterized, non-neutralizing head domain-specific antibody. For most adjuvanted groups, some competition was seen with the P1-05 anchor epitope mAb, which was either similar to or somewhat higher than that seen with the CR6261 central stem mAb. These might reflect the frequency of stem-binding antibodies, where anchor epitope mAbs generally are more frequent than central stem mAbs, although this has been shown for human but not mouse models [13]. The Y2+no adjuvant group, however, did appear to differ from all adjuvanted groups, showing no competition with CA09-38 and moderate competition with head-binding mAbs. The overall similarity in targeted epitopes across adjuvant groups may suggest a more general mode of enhancing the antibody response between them without much change to the overall frequencies of serum epitopes.

The functionality of serum antibodies following Y2 vaccination were assessed by HAI against a panel of recent H1N1 viruses. Whereas those against A/CA/09 were relatively high for all groups, they generally decreased somewhat against the A/BR/18 and A/GM/19 viruses. AddaS03 elicited the highest and broadest HAI activity, followed by Alhydrogel, and then AddaVax and CpG. Median titers remained above 1:80, our defined threshold for seroprotection, for all adjuvanted groups with Y2 against all viruses, whereas it dropped slightly below this threshold for the non-adjuvanted Y2 group for A/GM/19. The gradual decrease in relative HAI titers with more recent H1N1 viruses might reflect the effects of antigenic drift that permit some escape from antibodies against earlier 2009 pandemic-like viruses. However, with the addition of adjuvant, sufficiently high amounts of HAI-active antibodies may be induced that can overcome this effect.

The NA protein is more conserved than the HA protein across influenza viruses, making it an attractive candidate for a universal vaccine. Likewise, we assessed antibody responses to an N1 subtype NA COBRA, N1-I, when combined with adjuvant. Following a prime-boost regimen, titers followed the same general trend as for Y2, where AddaS03 elicited the highest titers against N1-I, followed by AddaVax, and then Alhydrogel and CpG. Differences between adjuvant groups, however, were not as pronounced as those seen with Y2, possibly because of the immunosubdominant nature of NA to HA [32]. Nonetheless, the trends observed between adjuvants persisted in NAI assays against the A/CA/09 2009 pandemic virus, with animals immunized with N1-I+AddaS03 showing significantly increased activity relative to the non-adjuvanted control. Interestingly, these trends between adjuvants were consistent between subcutaneous and intramuscular immunization between the H1 and N1 studies. It may be of interest to perform NAIs against more recent H1N1 isolates following N1-I immunization and assess whether activity decreases like that seen for HAIs against the A/BR/18 and A/GM/19 viruses. In general, these suggest that AddaS03 is the most efficacious adjuvant with respect to total IgG titers and NAI-based functionality.

Adjuvants are an integral component of several vaccines, including currently approved influenza vaccines like FluAd and Pandemrix [22]. These are tailored towards higher-risk populations, where the action of adjuvant is needed to induce a sufficiently protective immune response. In the development of universal COBRA vaccines, the use of adjuvant is likely needed to enhance the immune response. The selection of adjuvant has been known to alter antibody titers and the corresponding protection that is afforded. Therefore, in this study, we assayed a number of adjuvants that act through distinct mechanisms. We found that AddaS03 affords both the highest binding and functional antibody titers following Y2 and N1-I immunization. In addition, it elicited significant serum antibodies against the RBS epitope and the stem anchor epitope relative to other adjuvants. AddaS03, an analog of AS03, contains a number of immune-stimulating components that might prime the increased antibody response observed in this study. This includes DL-α-tocopherol, which has been shown to strengthen the innate immune response, and, consequently, the cell-mediated and humoral responses [33]. This adjuvant has also been associated with enhanced somatic hypermutation and affinity maturation that could account for the enhanced functional breadth following Y2 or N1-I immunization [34]. Importantly, in our study, we did not combine the Y2 and N1-I COBRAs in a single immunization, which may be of interest to investigate the effects of adjuvant in the context of HA and NA immunodominance. Nevertheless, our studies provide data on polyclonal antibody breadth, epitopes, and functionality that can inform adjuvant selection for COBRA candidates as they enter clinical studies.

## Data Availability

The data presented in this study are available on request from the corresponding author.

## Supplementary Materials

The following supporting information can be downloaded at: https://www.mdpi.com/article/10.3390/v15020347/s1, Figure S1: Competition ELISA plots of d56 Y2- and CA09-immunized mice sera against human mAbs for CA09 HA.

## Abbreviations

HA: hemagglutinin; NA, neuraminidase; IAV, influenza A virus; IBV, influenza B virus; cHA, chimeric hemagglutinin; mAb, monoclonal antibody; HAI, hemagglutination inhibition assay; NAI, neuraminidase inhibition assay.
