# Supplementary material for: Adjuvant-Mediated Differences in Antibody Responses to Computationally Optimized Hemagglutinin and Neuraminidase Vaccines
Source: Viruses. 2023 Jan 25;15(2):347. doi: 10.3390/v15020347 (PMC9960755; doi:10.3390/v15020347)
Supplement: Supplementary file 1 [file viruses-15-00347-s001.zip › viruses-2172276-supplementary.pdf]

Supplementary Figures

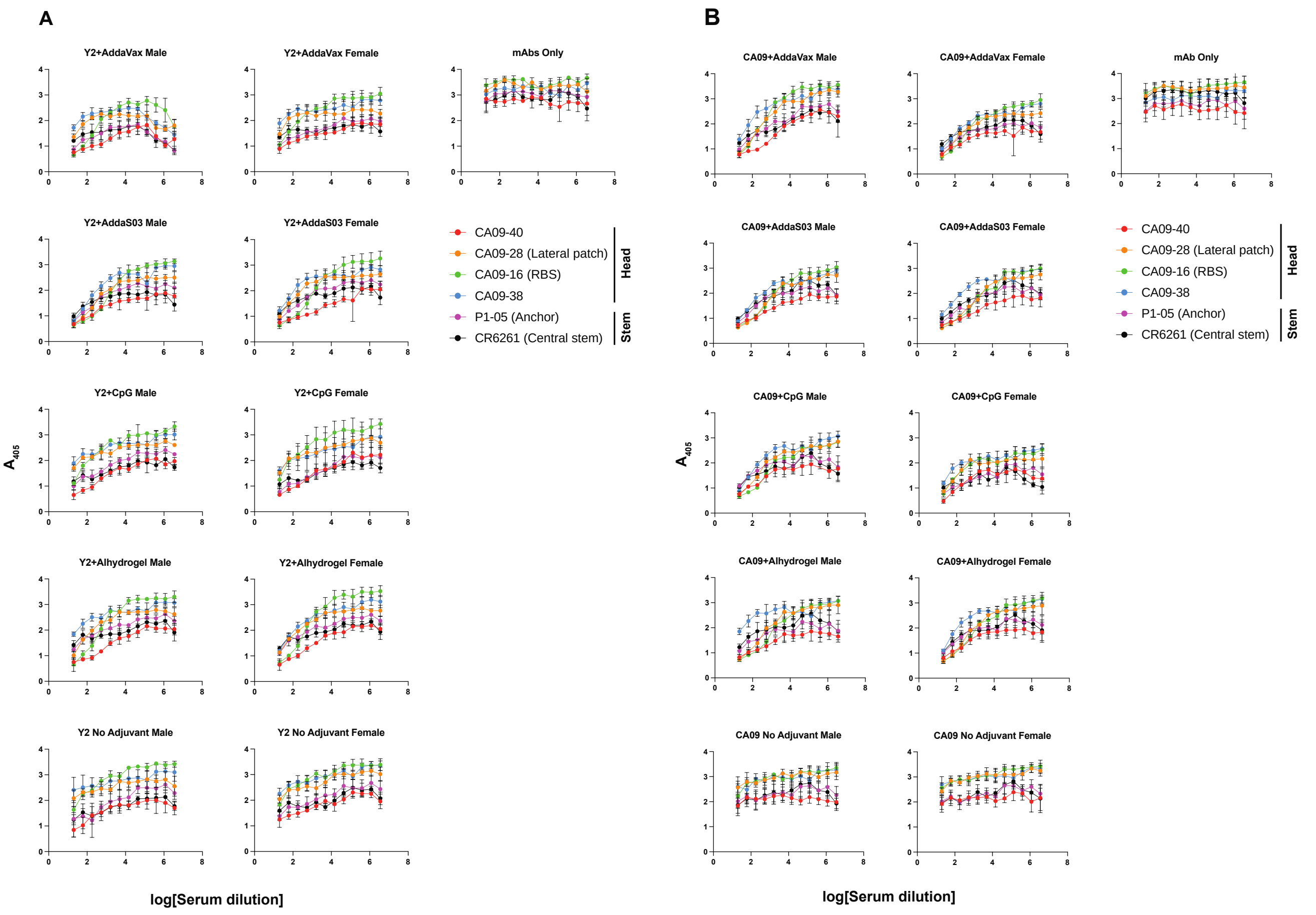

Figure S1. Competition ELISA plots of d56 Y2- and CA09-immunized mice sera against human mAbs for CA09 HA. Plots of serum competition with the six mAbs for pooled male and female mouse serum. Signal from the human mAb decreases with increasing competition from serum. The absorbance at 405 nm of each human mAb is shown for each dilution of pooled mice sera for (A) Y2- and (B) CA09-immunized animals.
